# Oral levodopa rescues retinal morphology and visual function in a murine model of human albinism

**DOI:** 10.1111/pcmr.12782

**Published:** 2019-04-02

**Authors:** Helena Lee, Jennifer Scott, Helen Griffiths, Jay E. Self, Andrew Lotery

**Affiliations:** ^1^ Clinical and Experimental Sciences, Faculty of Medicine Sir Henry Wellcome Laboratories, Southampton University Hospital University of Southampton Southampton UK; ^2^ Eye Unit University Hospital Southampton NHS Foundation Trust Southampton UK

**Keywords:** albinism, levodopa, neuronal plasticity, ocular, retina, therapeutics, vision

## Abstract

Albinism is a group of disorders characterized by pigment deficiency and abnormal retinal development. Despite being a common cause for visual impairment worldwide, there is a paucity of treatments and patients typically suffer lifelong visual disability. Residual plasticity of the developing retina in young children with albinism has been demonstrated, suggesting a post‐natal window for therapeutic rescue. L‐3, 4 dihydroxyphenylalanine (L‐DOPA), a key signalling molecule which is essential for normal retinal development, is known to be deficient in albinism. In this study, we demonstrate for the first time that post‐natal L‐DOPA supplementation can rescue retinal development, morphology and visual function in a murine model of human albinism, but only if administered from birth or 15 days post‐natal age.

**Significance**

In this study, we demonstrate for the first time that abnormal retinal development, morphology and visual function can be rescued post‐natally using oral L‐dihydroxyphenylalanine (L‐DOPA) supplementation, if administered during the critical period of neuroplasticity, in a murine model of human albinism. By modelling the known post‐natal period of plasticity seen in the retina in human albinism and rescuing visual function, we show that oral L‐DOPA replacement in human albinism shows promise as a therapeutic intervention during development.

## 1 INTRODUCTION

Albinism is a group of disorders of melanin biosynthesis that affects 1 in 4,000 people in the UK, and 1 in 1,000 people in sub‐Saharan Africa (Hong, Zeeb, & Repacholi, 2006; Sarvananthan et al., 2009). It can be divided into two subtypes: autosomal recessive oculocutaneous albinism (OCA), which manifests as hypopigmentation in the skin, hair and eyes, and X‐linked ocular albinism, which only manifests in the eyes. Additional ocular features include nystagmus, strabismus, refractive errors, abnormalities of retinal development (foveal hypoplasia) and optic nerve misrouting (Apkarian, 1992; Creel, Hendrickson, & Leventhal, 1982). The average visual acuity in OCA is reported to be 20/80, which is well below the UK minimum eyesight standard for driving (Cronin, Hertle, Ishikawa, & Schuman, 2009). However, visual acuity alone grossly underestimates the visual disorder which also includes photophobia (Digre & Brennan, 2012) and nystagmus (Chung, LaFrance, & Bedell, 2011) in most cases. Consequently, the majority of children with albinism are registered as sight impaired in the UK (Certificate of Visual Impairment, CVI) and require significant, lifelong visual support. Accordingly, albinism is the third most common cause for CVI registration in children in the UK (Bunce, Zekite, Wormald, & Bowman, 2017) and this is mirrored worldwide. Albinism also has significant effects on school performance, employment and quality of life, and carries a negative stigma especially in people of Southeast Asian and African descent (Kiprono, Joseph, Naafs, & Chaula, 2012; Maia, Volpini, dos Santos, & Rujula, 2015). To date, children with albinism have been deprived of an effective treatment. For these reasons, one of the top 10 research priorities in Childhood Onset Disorders, as determined by the Sight Loss and Vision Priority Setting Partnership in 2013, is: “Can a treatment be developed to improve vision for people with albinism?”

In order to develop a targeted and effective treatment for the visual disability associated with albinism, the role of the pigment synthesis pathway in normal ocular development needs to be interrogated (McKay, 2018). Normal ocular pigmentation and development are dependent upon tyrosinase (TYR) enzyme, (Beermann et al., 1990) which catalyses the conversion of tyrosine to L‐dihydroxyphenylalanine (L‐DOPA), phaeomelanin, eumelanin and dopamine (DA) (Hearing & Jimenez, 1987). DA both induces and limits neurite elongation in the developing retina, by coupling with D1 and D2 dopaminergic receptors, respectively (Eisenhofer et al., 2003). L‐DOPA also acts on OA1 (an orphan G‐protein‐coupled receptor) to upregulate pigment epithelium‐derived growth factor, a key regulator of retinal development (Lopez, Decatur, Stamer, Lynch, & McKay, 2008). This pathway is disrupted in albinism, resulting in foveal hypoplasia and visual impairment. It is hypothesized that retinal development arrests prematurely in albinism (McAllister et al., 2010; Wilson, Mets, Nagy, & Kressel, 1988). However, it has been demonstrated that retinal development is not arrested in young children with albinism, but is ongoing, albeit at a reduced rate and magnitude (Lee et al., 2015). This suggests that intervention(s), which take advantage of this post‐natal period of neuroplasticity, may promote normal retinal development and improve visual function in albinism. It is also notable that the onset of nystagmus in children with albinism is typically between 2 and 6 months of age, again suggesting a period of plasticity and potential therapeutic window in the developing visual system (Gottlob, 1997). This is a feasible concept, as albinism can be diagnosed in early infancy (Apkarian, 1992).

One possible intervention is oral L‐DOPA supplementation. In the developing murine retina, L‐DOPA levels increase throughout pre‐ and post‐natal development until the end of the first post‐natal month (Roffler‐Tarlov, Liu, Naumova, Bernal‐Ayala, & Mason, 2013). The post‐natal peak of L‐DOPA expression coincides with bipolar cell and rod photoreceptor development, and the peak of synaptogenesis in the murine retina (Xu & Tian, 2004). L‐DOPA is known to be deficient in the developing retina in murine OCA (Roffler‐Tarlov et al., 2013). Oral L‐DOPA replacement in pregnant albino mouse mothers results in the accumulation of L‐DOPA in the retinal pigment epithelium of the foetuses (Roffler‐Tarlov et al., 2013). Normally, L‐DOPA is synthesized from L‐tyrosine by two enzymes, tyrosinase (TYR) and tyrosine hydroxylase (TH). TYR is necessary for the subsequent conversion of L‐DOPA to melanin, within ocular melanosomes (Lavado, Jeffery, Tovar, de la Villa, & Montoliu, 2006). Ectopic expression of TH in the retinal pigment epithelium (RPE) in the absence of functioning TYR enzyme rescues the retinal abnormalities and visual function in albino mice in the absence of melanin synthesis (Lavado et al., 2006). This suggests that lack of L‐DOPA or one of its metabolic derivatives is key to the abnormal retinal development and function seen in albinism (Lavado et al., 2006). However, post‐natal modulation of retinal morphology and function using oral L‐DOPA in albinism has never been demonstrated and is key to translation of this potential therapeutic to human trials.

The aim of this study was to investigate whether post‐natal retinal development, morphology and visual function in albinism can be modulated and improved through oral L‐DOPA supplementation, if administered during the critical period of neuroplasticity, in a murine model of human OCA.

## 2 MATERIALS AND METHODS

All experiments were performed in accordance with the ARVO Statement for the use of animals in ophthalmic and vision research, in accordance with the NC3Rs ARRIVE guidelines, and after approval was obtained from Animal Welfare and Ethical Review Body, University of Southampton (project licence number: P474F5E4D) (“ARVO Statement for the use of animals in ophthalmic & vision research”; NC3Rs Reporting Guidelines Working Group, 2010). All possible steps were taken to avoid animal suffering at each stage of the experiment.

### 2.1 Animals

The effects of oral L‐DOPA supplementation on retinal morphology and function were determined using optical coherence tomography (OCT) and electroretinography (ERG), respectively, in the C57BL/6J‐c2J null mouse model of OCA1 (CALBs) and C57BL/6J pigmented (B6) mice (Tables 1 and 2). The C57BL/6J‐c2J null mouse model of OCA1 (CALBs) was chosen for this study as it is established as a model for testing potential treatments for OCA (Reis, Ventura, Kubrusly, de Mello, & de Mello, 2007; Roffler‐Tarlov et al., 2013). Mice were housed under controlled light conditions (12:12 hr of light/dark cycle) with food and water available ad libitum. Animals (in each group) were examined at the age of 4, 5, 6, 8, 12 and 16 weeks post‐natal age (PNA). For ERG and OCT, animals were anesthetized with an intraperitoneal injection of a mixture of ketamine (100 mg/kg, Chanelle) and xylazine (10 mg/kg, Bayer) in 0.1 ml of saline. A total of 436 and 398 mixed cross‐sectional and longitudinal examinations were obtained from the CALBs and the B6 mice, respectively, at 4, 5, 6, 8, 12 and 16 weeks PNA. The authors, who prepared and administered the L‐DOPA treatment to the mice, also performed and analysed the OCT and ERG examinations.

**Table 1:** Summary of the numbers of mice included in each of the treatment arms

Treatment group	C57BL/6J‐c2J OCA	C57BL/6J pigmented mice
Controls	11	11
L‐DOPA from birth	11	9
L‐DOPA from 15 days PNA	8	11
L‐DOPA from 28 days PNA	10	5

**Table 2:** Summary of the L‐DOPA dosage and route of administration used for each of the three treatment time points

Time point	L‐DOPA dosage and route of administration
Birth	Preweaning: 1 mg/ml via the mouse mothers’ milk (3.3 mg/ml dissolved in drinking water to give an L‐DOPA concentration in breast milk of 1 mg/ml)[Link] Post‐weaning: 1 mg/ml dissolved in drinking water
Post‐natal day 15	
Post‐natal day 28	1 mg/ml dissolved in drinking water

Note

^a^Based on an L‐DOPA milk/plasma concentration of 0.3 (Witkovsky, 2004).

### 2.2 Treatment of mice with L‐DOPA

Albino and pigmented mice were supplemented with 1 mg/ml L‐DOPA (Sigma) dissolved in the drinking water with ascorbate (2.5 mg/ml to prevent oxidation) for 28 days, at three different time points (Birth, 15 and 28 days post‐natal age [PNA]), as per Table 2.

### 2.3 Electroretinogram recordings (ERG)

Electroretinogram recordings were performed with the Phoenix Micron III image‐guided focal ERG system designed for rodents. Mice were dark adapted for overnight and subsequently handled under dim red light illumination. Pupils were dilated using tropicamide 1% eye drops (Bausch & Lomb) and phenylephrine hydrochloride 2.5% eye drops (Bausch & Lomb), and corneas lubricated with a coupling gel (Clinitas gel, Altacor). Animals were positioned on a heated pad to maintain constant body temperature. Subdermal electrodes were used, with the ground electrode inserted in the lumbar region, and the reference electrode placed between the eyes. Scotopic central field focal ERGs were conducted by projecting a 1.5‐mm (approximately 7.7 disc diameters) circular LED white light stimulus diameter, at a flash strength of 6.8 log cd‐s/m^2^ on to the central retina white LED light for 1ms. Two sweeps per eye were carried out with an interval time of 120 s, from which average A‐ and B‐wave amplitudes and implicit times were calculated.

### 2.4 Optical coherence tomography (OCT)

Mice were anesthetized, and their pupils dilated as described above. Artificial tears (Systane Ultra, Alcon) were used throughout the procedure to maintain corneal moisture and clarity. Animals were wrapped in surgical gauze and positioned comfortably within a stereotactic rotational cassette, with their heads aligned on a bite bar, in an animal imaging mount. The Leica Envisu R2200 VHR SDOIS Mouse Imaging System was used to obtain 1.4 mm volumetric scans (consisting of 100 B‐scans and 1,000 A‐scans per B‐scan), centred on the optic disc.

Segmentation of the individual retinal layers was carried out at 24 retinal locations (238 micron increments) using InVivoVue 2.4 Diver software (Figure 3). Previously, the majority of OCT retinal findings in albinism have been described in humans, where albinism mainly affects macular morphology, a structure that is not present in the rodent eye (Mohammad et al., 2011). However, it has been demonstrated that there is an increased density of rod and cone photoreceptors in the central mouse retina, with a comparable rod to cone ratio between the central mouse retina and human macula at eccentricities between 10° and 20° (Volland, Esteve‐Rudd, Hoo, Yee, & Williams, 2015). Therefore, we focused our analysis on the points located within a 20° eccentricity in order to make our OCT analysis of the mouse central retina comparable to findings reported in the human macula (Figure 3).

### 2.5 Statistics and modelling

A linear mixed model, implemented in STATA™ (Copyright 1996–2014, StataCorp), was used to analyse the differences between untreated albino (CALBs) and pigmented (B6) mice with regard to A‐ and B‐wave amplitudes, A‐ and B‐wave latencies and thickness measurements obtained for each retinal layer. The model included fixed effects for mouse strain, age, eye and the interaction between mouse strain and age (mouse × age). The covariance structure was independent. All models included a random intercept and slope for each mouse thereby accounting for repeated measures over time. Post hoc estimates of the marginal effects of mouse strain on A‐ and B‐wave amplitudes, A‐ and B‐wave latencies and retinal layer thickness measurements for each retinal layer were calculated.

A separate linear mixed model was constructed for the CALBs and B6 mice, in order to identify the effects of altering the time of commencement of oral L‐DOPA supplementation on A‐ and B‐wave amplitudes, A‐ and B‐wave latencies and thickness measurements obtained for each retinal layer. The model included fixed effects for treatment group, age, eye and the interaction between treatment group and age (treatment × age). The covariance structure was independent. All models included a random intercept and slope for each mouse thereby accounting for repeated measures over time. Post hoc estimates of the marginal effects of mouse strain on A‐ and B‐wave amplitudes, A‐ and B‐wave latencies and retinal layer thickness measurements for each retinal layer were calculated.

To determine the statistical significance of a predictor or interaction term in each model, we considered type 1 error rate as <0.05 (*p* < 0.05).

Spearman's rank correlation was conducted in order to assess the relationship between retinal layer OCT thickness measurements and A‐ and B‐wave ERG amplitudes, using a Bonferroni correction for multiple comparisons.

## 3 RESULTS AND DISCUSSION

### 3.1 Oral L‐DOPA is tolerated well in treated mice

L‐dihydroxyphenylalanine is known to be deficient in the developing retina in OCA (Roffler‐Tarlov et al., 2013). In this study, we have demonstrated for the first time that it is possible to modulate post‐natal retinal morphology and rescue function and in albino (CALBs) mice, through the administration of a 28‐day course of oral L‐DOPA. Oral L‐DOPA supplementation at the dosages administered in this study was tolerated very well, with no signs of toxicity (e.g., erect tails, piloerection, ataxia, lacrimation, increased activity, clonic convulsions, increased irritability and head and body tremors) observed in any of the treated mice.

### 3.2 Post‐natal L‐DOPA supplementation improves retinal function in albino mice but not B6/control mice

**Figure 1:**
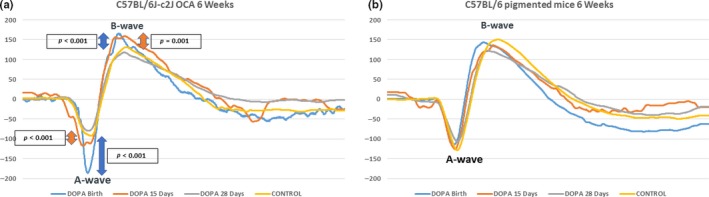
Example mean electroretinogram recordings from CALBs (a) and B6 (b) mice supplemented with L‐DOPA 1 mg/ml from birth, 15 and 28 days PNA, in comparison with untreated controls, at 6 weeks PNA. A more negative A‐wave amplitude (reflecting photoreceptor function) and a more positive B‐wave amplitude (reflecting inner retinal function) reflect better retinal function. There were significant (*p* < 0.001) increases in the A‐ and B‐wave amplitudes of the CALBs mice supplemented with L‐DOPA from birth and 15 days PNA in comparison with untreated controls. B6, C57BL/6 pigmented mice; CALBs, C57BL/6J‐c2J OCA mice; OCA, oculocutaneous albinism; PNA, post‐natal age

Electroretinography (ERG) is an established non‐invasive method to evaluate retinal function (Pinto, Invergo, Shimomura, Takahashi, & Troy, 2007). The amplitude of the electronegative A‐wave and electropositive B‐wave on ERG reflects photoreceptor (Penn & Hagins, 1969) and inner retinal (i.e., rod‐bipolar cell) (Robson, Maeda, Saszik, & Frishman, 2004) function, respectively. The effects of a 28‐day course of oral L‐DOPA supplementation (1 mg/ml dissolved in drinking water) administered to C57BL/6J‐c2J OCA mice (CALBs) from birth (*n* = 11), 15 days (*n* = 8) and 28 days (*n* = 10) post‐natal age (PNA) on retinal function were investigated using ERG (Phoenix Micron III), and compared to 11 untreated CALBs (Figure 1a). Similarly, ERGs recorded from C57BL/6 pigmented (B6) mice, also treated with 28 days of oral L‐DOPA supplementation from birth (*n* = 9), 15 days (*n* = 11) and 28 days (*n* = 5) PNA, were compared to 11 untreated B6 mice (Figure 1b). A total of 436 and 398 mixed cross‐sectional and longitudinal recordings were taken from the CALBs and the B6s, respectively, at 4, 5, 6, 8, 12 and 16 weeks PNA.

**Figure 2:**
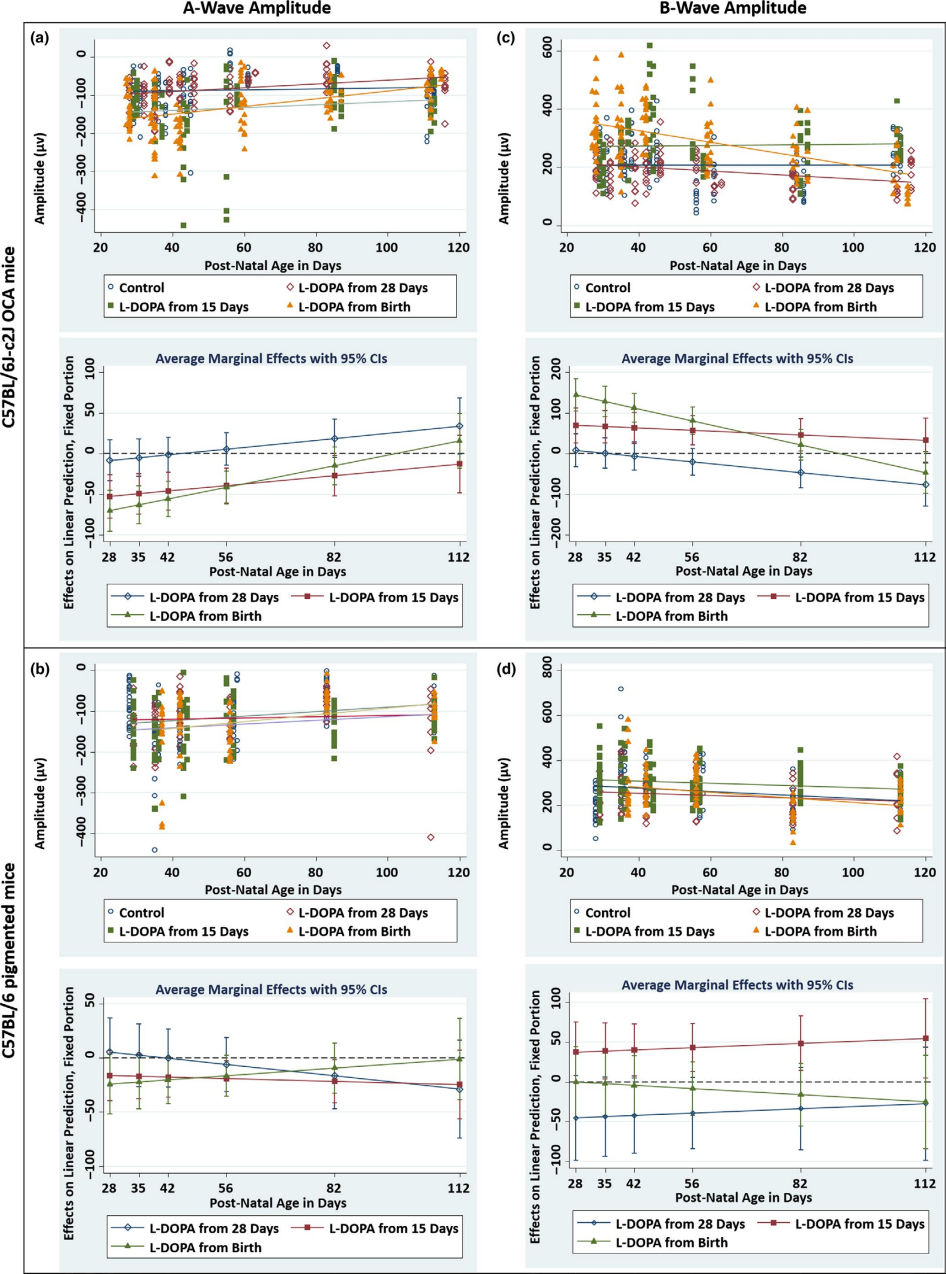
Electroretinogram results summarizing the A‐ (a) & (b) and B‐wave (c) & (d) amplitudes recorded from CALBs (a) & (c) and B6 (b) & (d) mice treated with L‐DOPA from birth, 15 and 28 days PNA, in comparison with untreated controls. The upper plots for each panel show the A‐ and B‐wave amplitudes plotted with respect to post‐natal age. Each point represents a single value from each ERG examination. The lines of best fit (trend lines) are shown in navy for the control mice, and maroon, green and orange for the mice treated from 28 days PNA, 15 days PNA and birth, respectively. The lower plots for each panel are marginal effects plots summarizing the average differences in A‐ and B‐wave amplitudes, between each of the three treatment groups in comparison to untreated controls (*y* = 0), with respect to post‐natal age. The error bars are representing the 95% confidence intervals. By calculating partial derivatives of the interaction term from the linear mixed model, the significant differences between each of the three treatment groups and the control groups were estimated at six specified time points: 28, 35, 42, 56, 82 and 112 days PNA. B6, C57BL/6 pigmented mice; CALBs, C57BL/6J‐c2J OCA mice; ERG, electroretinogram; OCA, oculocutaneous albinism; PNA, post‐natal age

A‐ (*z* = −2.85, *p* = 0.004) and B‐wave (*z* = 3.97, *p* < 0.001) amplitudes were significantly greater in untreated pigmented (B6) mice, in comparison with untreated albino (CALBs) mice (see Table 3 for mean values). L‐DOPA supplementation did not significantly alter A‐ or B‐wave amplitudes in B6 mice (Figures 1b and 2b,d). However, both A‐ and B‐wave amplitudes were significantly increased in CALBs supplemented with L‐DOPA from birth (A‐wave: *z* = −5.5, *p* < 0.001 & B‐wave: *z* = 7.6, *p* < 0.001) and 15 days PNA (A‐wave: *z* = −3.44, *p* = 0.001 & B‐wave: 2.79, *p* = 0.005), but not in CALBs treated after 28 days PNA (Table 3). Interestingly, this therapeutic window corresponds with the post‐natal peak of L‐DOPA expression (25.5 days PNA) (Roffler‐Tarlov et al., 2013) and synaptogenesis in the developing mouse eye. However, this effect was not sustained beyond 3 months PNA, suggesting that a longer course of L‐DOPA supplementation or possibly lifelong therapy may be needed in order to achieve a sustained improvement in retinal function (Figure 2a,c).

**Table 3:** Summary of the means, standard deviations and linear mixed model results for each of the ERG and OCT parameters assessed with respect to each of the three L‐DOPA treatment groups (28 days of treatment from birth, 15 or 28 days post‐natal age) in comparison with untreated controls, for both albino (a) and pigmented (b) mice

	Control	L‐DOPA from birth			L‐DOPA from 15 days			L‐DOPA from 28 days		
	Mean (*SD*)	Mean (*SD*)	*z*	*p*	Mean (*SD*)	*z*	*p*	Mean (*SD*)	*z*	*p*
a. CALBs										
A‐wave amplitude	−86.45 (51.83) µV	**−132.36 (64.57) µv**	**−5.5**	**<0.001**	**−132.71 (75.44) µv**	**−3.44**	**0.001**	−83.02 (44.52) µV	−1.22	0.221
B‐wave amplitude	207.65 (77.98) µV	**290.81 (111.35) µv**	**7.6**	**<0.001**	**275.00 (102.01) µv**	**2.79**	**0.005**	189.65 (58.29) µV	1.3	0.192
A‐wave latency	11.27 (7.5) ms	**12.22 (4.03) ms**	**−2.64**	**0.008**	**12.96 (5.01) ms**	**−2.13**	**0.033**	11.12 (3.68) ms	−1.09	0.274
B‐wave latency	49.5 (11.5) ms	46.85 (17.15) ms	−0.82	0.414	49.18 (12.46) ms	1.4	0.162	49.08 (13.47) ms	0.21	0.832
RNFL thickness	7.6 (1.1) µm	**9.89 (2.01) µm**	**2.01**	**0.044**	**12.23 (1.18) µm**	**6.52**	**<0.001**	8.01 (1.82) µm	−0.5	0.614
GCL‐IPL thickness	52.1 (3.2) µm	51.15 (3.65) µm	1.92	0.055	49.68 (2.26) µm	0.79	0.428	**49.95 (3.48) µm**	**−2.88**	**0.004**
INL thickness	26.4 (1.8) µm	25.96 (1.94) µm	1.3	0.194	**26.61 (2.02) µm**	**2.3**	**0.022**	**25.62 (1.55) µm**	**−2.45**	**0.014**
OPL thickness	9.3 (1.2) µm	**9.87 (1.09) µm**	**−2.08**	**0.038**	**11.11 (0.83) µm**	**3.6**	**<0.001**	**10.33 (2.11) µm**	**2.47**	**0.013**
ONL thickness	60.4 (2.6) µm	**59.34 (2.77) µm**	**1.97**	**0.049**	**59.58 (2.76) µm**	**3.9**	**<0.001**	**58.77 (3.28) µm**	**−2.29**	**0.022**
IS thickness	14.1 (1.4) µm	13.95 (1.46) µm	0.39	0.696	13.70 (1.31) µm	−0.81	0.42	13.95 (1.55) µm	1.19	0.232
OS thickness	6.9 (0.89) µm	**7.68 (1.21) µm**	**−2.32**	**0.02**	**9.45 (0.64) µm**	**5.98**	**<0.001**	7.28 (1.26) µm	1.76	0.078
ETPRS thickness	20.44 (3.89) µm	**18.30 (2.45) µm**	**3.43**	**0.001**	**17.92 (1.75) µm**	**3.97**	**<0.001**	19.62 (3.08) µm	1.68	0.092
RPE Thickness	6.5 (1.2) µm	**6.95 (0.82) µm**	−1.75	0.08	**8.08 (0.66) µm**	**2.4**	**0.016**	7.04 (1.59) µm	1.6	0.11
b. B6 mice										
A‐wave amplitude	−113.03 (66.51) µV	−124.62 (66.10) µv	−1.48	0.138	−131.62 (58.48) µv	−0.78	0.436	−117.03 (66.01) µv	0.72	0.473
B‐wave amplitude	261.77 (98.91) µV	255.75 (95.14) µv	0.24	0.81	297.24 (90.33) µv	1.15	0.25	244.39 (96.51) µv	−1.4	0.161
A‐wave latency	15.7 (9.7) ms	14.70 (4.93) ms	0	0.997	**14.10 (4.25) ms**	**−2.48**	**0.013**	15.24 (4.87) ms	1.31	0.189
B‐wave latency	54.7 (17.6) ms	49.50 (15.59) ms	−1.15	0.248	47.98 (11.34) ms	0.24	0.809	**57.08 (19.53) ms**	**2.21**	**0.027**
RNFL thickness	9.8 (1.9) µm	9.21 (1.61) µm	1.64	0.1	**12.29 (1.35) µm**	**5.7**	**<0.001**	**9.47 (1.87) µm**	**2.11**	**0.035**
GCL‐IPL thickness	50.7 (3.3) µm	**51.12 (3.66) µm**	**−4.07**	**<0.001**	50.10 (2.39) µm	−0.74	0.457	**50.62 (4.02) µm**	**−4.59**	**<0.001**
INL thickness	25.5 (1.6) µm	25.22 (1.31) µm	−1.62	0.105	**26.26 (1.69) µm**	**4.43**	**<0.001**	**24.88 (1.74) µm**	−0.05	0.963
OPL thickness	10.4 (1.4) µm	10.45 (1.26) µm	0.87	0.383	10.87 (1.00) µm	0.14	0.885	**10.69 (1.93) µm**	**3.82**	**<0.001**
ONL thickness	59.5 (1.8) µm	**59.57 (2.15) µm**	**−3.55**	**<0.001**	**60.14 (1.96) µm**	**2.86**	**0.004**	60.21 (2.18) µm	−1.94	0.052
IS thickness	12.2 (1.3) µm	**12.66 (1.12) µm**	**2.6**	**0.009**	**12.86 (1.02) µm**	**5.16**	**<0.001**	**12.98 (1.97) µm**	**8.45**	**<0.001**
OS thickness	7.9 (0.87) µm	7.75 (0.83) µm	1.59	0.112	**9.08 (0.73) µm**	**6.01**	**<0.001**	**7.73 (1.20) µm**	**2.06**	**0.04**
ETPRS thickness	18.97 (2.46) µm	19.52 (2.30) µm	0.15	0.884	**21.14 (1.28) µm**	**10.77**	**<0.001**	18.85 (2.12) µm	1.12	0.262
RPE thickness	9.8 (1.2) µm	9.39 (1.25) µm	0.34	0.731	**9.58 (0.57) µm**	**−3.56**	**<0.001**	9.28 (1.56) µm	0.01	0.995

Note

B6: C57BL/6 pigmented mice; CALBs: C57BL/6J‐c2J OCA mice; ERG: electroretinogram; ETPRS: photoreceptor end tips; GCL‐IPL: combined ganglion cell‐inner plexiform layer complex; INL: inner nuclear layer; IS: photoreceptor inner segment; OCT: optical coherence tomography; ONL: outer nuclear layer; OPL: outer plexiform layer; OS: photoreceptor outer segment; RNFL: retinal nerve fibre layer; RPE: retinal pigment epithelium.

Results highlighted in bold are statistically significant.

There were no significant differences in A‐ and B‐wave latencies, between untreated B6 mice and untreated CALBs (see Table 3 for mean values). A‐wave latencies were significantly decreased in CALBs supplemented with L‐DOPA from birth (*z* = −2.64, *p* = 0.008) and 15 days PNA (*z* = −2.13, *p* = 0.033), but not in CALBs treated after 28 days PNA (Table 3). L‐DOPA treatment became less effective with increasing age. Interestingly, significant decreases in A‐wave latencies were also observed in B6 mice treated from 15 days PNA (*z* = −2.48, *p* = 0.013), but not in B6 mice treated from birth or 28 days PNA.

B‐wave latencies decreased with age in both CALBs (*z* = −2.4, *p* = 0.017) and B6 mice (*z* = −2.46, *p* = 0.014) treated with L‐DOPA from 15 days PNA, resulting in significant reductions from 3 months and 6 weeks PNA, respectively. In contrast, B‐wave latencies were significantly increased in B6 mice treated from 28 days PNA (*z* = 2.21, *p* = 0.027) (Table 3). B‐wave latencies were not significantly altered in CALBs and B6 mice treated from birth or CALBs treated from 28 days PNA.

It is known that L‐DOPA supplementation increases dopamine (DA) levels in both the pigmented and albino mouse neural retina, through the conversion of DOPA to DA by intrinsic aromatic amino acid decarboxylase. DA has both synaptic and paracrine actions within the neural retina, which includes the modulation of photoreceptor activity and enhancement of light‐driven cone input (Masson, Mestre, & Blin, 1993; Witkovsky, 2004). Potentially, L‐DOPA supplementation could trigger reductions in A‐ and B‐wave latencies on ERG recordings, through the enhancement of dopaminergic signalling within the neural retina (Schorderet & Nowak, 1990; Witkovsky, 2004). In the next phase of our work, we are planning to compare these ERG results, to visual acuity results obtained by assessing the optomotor response in awake mice, for the purposes of future translational research studies (Prusky & Douglas, 2004).

### 3.3 Post‐natal L‐DOPA supplementation increases retinal nerve fibre layer (RNFL) thickness in albino and pigmented mice

**Figure 3:**
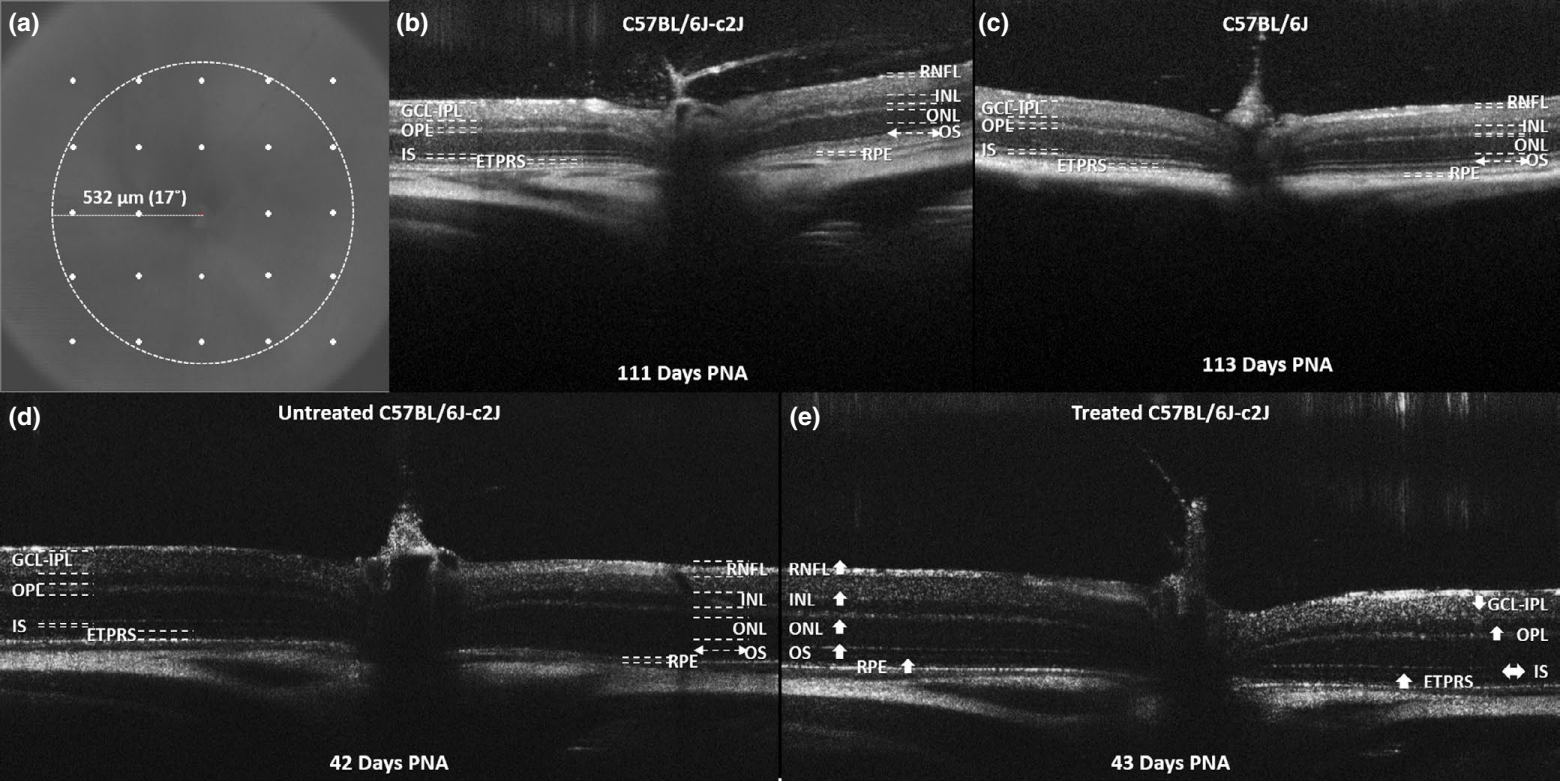
Example of in vivo optical coherence tomography (OCT) retinal imaging obtained from untreated C57BL/6J‐c2J (CALB) (b) & (d) and C57BL/6 pigmented (B6) mice (c) and a CALB mouse supplemented with a 28‐day course of oral L‐DOPA from 15 days PNA (e). The en fosse view (a) is shown with the 24 segmentation locations surrounding the optic nerve highlighted in white. The circle is highlighting the central 17° retinal area surrounding the optic nerve, where the OCT segmentation analysis was focused. The arrows in (e) indicate the effects or oral L‐DOPA supplementation on each of the individual retinal layer thicknesses in comparison with untreated CALBs. The up and down arrows are indicating significant increases and decreases, respectively, in retinal layer thickness measurements. The lack of pigment in the CALB retina facilitates deeper penetration of the OCT through the RPE, artificially creating the appearance of a thicker choroid. B6, C57BL/6 pigmented mice; CALBs, C57BL/6J‐c2J OCA mice; ETPRS, photoreceptor end tips; GCL‐IPL, combined ganglion cell‐inner plexiform layer complex; INL, inner nuclear layer; IS, photoreceptor inner segment; OCA, oculocutaneous albinism; ONL, outer nuclear layer; OPL, outer plexiform layer; OS, photoreceptor outer segment; RNFL, retinal nerve fibre layer; RPE, retinal pigment epithelium

Optical coherence tomography (OCT) is a non‐invasive imaging method that provides high‐resolution cross‐sectional images of in vivo retinal morphology (Adachi et al., 2016; Gabriele et al., 2011). Retinal OCT imaging has been correlated with histological, ultrastructural and electroretinography findings and has the significant advantage of facilitating longitudinal evaluation of disease progression (Adachi et al., 2016; Spaide & Curcio, 2011; Vajzovic et al., 2012). The effects of a 28‐day course of oral L‐DOPA supplementation on in vivo retinal development and morphology were investigated using OCT imaging (Leica Envisu R2200 Mouse Imaging System) (Figure 3).

Retinal nerve fibre layer thickness measurements were significantly greater in untreated pigmented (B6) mice, in comparison with untreated albino (CALBs) mice (*z* = 2.14, *p* = 0.032) (see Table 3 for mean values). We observed significant increases in RNFL thickness measurements in CALBs supplemented with L‐DOPA from birth (*z* = 2.01, *p* = 0.044) and 15 days PNA (*z* = 6.52, *p* < 0.001), and B6 mice supplemented from 15 (*z* = 5.7, *p* < 0.001) and 28 days PNA (*z* = 2.11, *p* = 0.035) (Table 3, Figure S1). L‐DOPA had no effect on RNFL thickness measurements in CALBs treated after 28 days PNA. Treatment appeared to have greater effects with increasing age in CALBs supplemented from birth (*z* = 2.36, *p* = 0.018) (Figure S1). In contrast, treatment became less effective with increasing age in B6 mice supplemented from birth (*z* = −2.91, *p* = 0.004) and 28 days PNA (*z* = −3.52, *p* < 0.001) (Figure S1).

### 3.4 L‐DOPA supplementation decreases the combined ganglion cell layer‐inner plexiform layer (GCL‐IPL) thickness in albino and pigmented mice

The GCL‐IPL thickness measurements were significantly greater in untreated albino (CALBs) mice, in comparison with untreated pigmented (B6) mice (*z* = 2.58, *p* = 0.010) (see Table 3 for mean values). GCL‐IPL thickness measurements were significantly decreased in CALBs supplemented with L‐DOPA from 28 days PNA (*z* = −2.88, *p* = 0.004) and B6 mice supplemented from birth (*z* = −4.07, *p* < 0.001) and 28 days PNA (*z* = −4.59, *p* < 0.001) (Table 3, Figure S1). Significant decreases in GCL‐IPL thickness measurements also became evident with increasing age, in CALBs treated with L‐DOPA from birth (*z* = −2.6, *p* = 0.009) and 15 days PNA (*z* = −2.39, *p* = 0.017) (Figure S1). This suggests that L‐DOPA supplementation decreases retinal ganglion cell differentiation rates in both CALBs and B6 mice, resulting in decreased GCL‐IPL thickness measurements (Young, 1984). Alternatively, this may be reflecting enhancements in retinal ganglion cell dendritic development and pruning in the L‐DOPA supplemented mice (Tian, 2011). In contrast, the treatment became less effective with increasing age in B6 mice treated from birth (*z* = 4.49, *p* < 0.001) and 28 days PNA (*z* = 4.15, *p* < 0.001) (Figure S1). There were no significant differences in GCL‐IPL thickness measurements in B6 mice treated from 15 days PNA.

### 3.5 L‐DOPA supplementation increases inner nuclear layer (INL) thickness in albino and pigmented mice

There were no significant differences in INL thickness measurements between untreated pigmented (B6) and albino (CALBs) mice (Table 3). INL thickness measurements were significantly increased in CALBs (*z* = 2.3, *p* = 0.022) and B6 mice (*z* = 4.43, *p* < 0.001) supplemented with L‐DOPA from 15 days PNA, and decreased in CALBs supplemented from 28 days PNA (*z* = −2.45, *p* = 0.014) (Table 3, Figure S1). Significant decreases in INL thickness measurements also became evident with increasing age, in B6 mice treated from 28 days PNA (*z* = −2.00, *p* = 0.046) (Figure S1). These observations may be consistent with previously reported descriptions of the patterns of retinal cell addition and production in the INL of albino rats (Ilia & Jeffery, 2000). In these studies, it has been demonstrated that cellular proliferation is significantly elevated in the INL of albino rats, in comparison with pigmented rats, at the majority of time points between post‐conception days (PCD) 14 and 28, with consequential transient thickening of the INL. This is then followed by a wave of excessive cell death around the time of birth, which depletes cell numbers and consequently eliminates the differences in INL thickness measurements between albino and pigmented rats (Ilia & Jeffery, 2000). Perhaps, L‐DOPA supplementation *after* the majority of cells in the INL have been produced, but during the time period when excessive cell death is expected to occur, is reducing cell death by stabilizing retinal mitosis. This would explain the transient increase in INL thickness observed in this particular treatment group. Conversely, supplementation at a later time point (28 days PNA) results in a decrease in INL thickness measurements in CALBs and B6 mice, by decreasing the degree of cell proliferation that is occurring, after a significant proportion of cell death has already occurred. This highlights how altering the timing of L‐DOPA supplementation can result in different retinal morphological outcomes. There were no significant differences in INL thickness measurements in CALBs and B6 mice treated from birth.

### 3.6 L‐DOPA supplementation increases outer plexiform layer (OPL) thickness in albino and pigmented mice

Outer plexiform layer thickness measurements were significantly greater in untreated pigmented (B6) in comparison with untreated albino (CALBs) mice (*z* = 3.25, *p* = 0.001) (see Table 3 for mean values). In the OPL, cone pedicles and rod spherules are synaptic upon various bipolar cell and horizontal cell types. It has previously been demonstrated that there is a failure of 30% of the rod population to develop in albino mice (Ilia & Jeffery, 2000). Given this reduction in rod numbers, there is likely to be a decrease in the number of synapses between rod photoreceptors, horizontal and bipolar cells in the OPL, which could potentially manifest as a reduction in OPL thickness measurements in the CALBs (Ilia & Jeffery, 2000). OPL thickness measurements were significantly increased in CALBs treated from 15 (*z* = 3.6, *p* < 0.001) and 28 days PNA (*z* = 2.47, *p* = 0.013), and B6 mice supplemented from 28 days PNA (*z* = 3.82, *p* < 0.001) (Table 3, Figure S2). Interestingly, although OPL thickness measurements were initially decreased in CALBs treated from birth, significant increases emerged with increasing age (*z* = 3.93, *p* < 0.001) (Figure S2). In contrast, significant decreases in OPL thickness measurements emerged with increasing age, in B6 mice treated from 28 days PNA (*z* = −3.86, *p* < 0.001) (Figure S2). This suggests that L‐DOPA supplementation in CALBs is potentially increasing the number of rod photoreceptors together with their associated synapses in the OPL, ultimately manifesting as an increase in OPL thickness measurements. However, this increase appears to be only sustained in CALBs treated before 28 days PNA, that is, during the period when L‐DOPA levels normally peak in the developing mouse eye (Roffler‐Tarlov et al., 2013). There were no significant differences in OPL thickness measurements in B6 mice treated from birth and 15 days PNA.

### 3.7 L‐DOPA supplementation increases outer nuclear layer (ONL) thickness in albino and pigmented mice

There were no significant differences between ONL thickness measurements in untreated pigmented (B6) and albino (CALBs) mice (see Table 3 for mean values). ONL thickness measurements were significantly increased in CALBs supplemented with L‐DOPA from birth (*z* = 1.97, *p* = 0.049) and 15 days PNA (*z* = 3.9, *p* < 0.001), and B6 mice supplemented from 15 days PNA (*z* = 2.86, *p* = 0.004) (Table 3, Figure S2). In contrast, ONL thickness measurements were significantly decreased in CALBs supplemented from 28 days PNA (*z* = −2.29, *p* = 0.022) (Table 3, Figure S2). Treatment became less effective with increasing age, until a tendency towards significantly decreased ONL thickness measurements emerged in CALBs treated from birth (*z* = −3.15, *p* = 0.002) and 15 days PNA (*z* = −5.2, *p* < 0.001) (Figure S2). This suggests that L‐DOPA supplementation during the expected peak of post‐natal DOPA expression can increase rod photoreceptor numbers in albinism, although this effect is not sustained (Ilia & Jeffery, 2000). There were no significant differences in ONL measurements in B6 mice treated from birth and 28 days PNA.

### 3.8 Photoreceptor inner segment (IS) length is unaffected in albino and increased in pigmented mice by L‐DOPA supplementation

Inner segment length measurements were significantly lower in untreated pigmented (B6) mice in comparison with untreated albino (CALBs) mice (*z* = −6.80, *p* < 0.001) (see Table 3 for mean values). The IS normally forms the junction between the OS and the synapse, and the reduction in IS length measurements in untreated CALBs may reflect either a reduction in the number of rod photoreceptors or defective synaptogenesis, the peak of which normally coincides with the peak of DOPA expression in the developing mouse eye (Xu & Tian, 2004). Unexpectedly, there were no significant differences in IS length measurements in CALBs supplemented with L‐DOPA from birth, 15 or 28 days PNA (Table 3, Figure S2). Conversely, IS length measurements were significantly increased in B6 mice supplemented with L‐DOPA from birth (*z* = 2.6, *p* = 0.009), 15 (*z* = 5.16, *p* < 0.001) and 28 days PNA (*z* = 8.45, *p* < 0.001) (Table 3, Figure S2). Treatment became less effective with increasing age, until a tendency towards significantly reduced IS length measurements emerged in B6 mice treated from 28 days PNA (Figure S2). If L‐DOPA treatment increases the number of rod photoreceptors and their associated synapses in albinism, then one might expect to see this manifest morphologically, as an increase in photoreceptor IS length measurements in treated mice. Perhaps as L‐DOPA is being administered towards the end of the developmental period, there is not a large enough effect on rod photoreceptor numbers in order for it to manifest as a significant change in photoreceptor IS length measurements on OCT examinations.

### 3.9 L‐DOPA supplementation increases photoreceptor outer segment (OS) length in albino and pigmented mice

There were no significant differences between photoreceptor outer segment (OS) length measurements recorded from untreated pigmented (B6) mice and untreated albino (CALBs) mice (see Table 3 for mean values). OS length measurements were significantly increased in CALBs supplemented with L‐DOPA from 15 days PNA (*z* = 5.98, *p* < 0.001), but not in CALBs treated after 28 days PNA (Table 3, Figure S3). Although OS length measurements were initially decreased in CALBs treated from birth, significant increases emerged with increasing age (*z* = 5.29, *p* < 0.001) (Figure S3). The OS length measurements were also significantly increased in B6 mice treated from 15 (*z* = 6.01, *p* < 0.001) and 28 days PNA (*z* = 2.06, *p* = 0.04) (Table 3, Figure S3). Treatment became less effective with increasing age, until a tendency towards significantly decreased OS length measurements emerged in B6 mice treated from birth, 15 and 28 days PNA (Figure S3). L‐DOPA upregulates pigment epithelium‐derived growth factor (PEDF), as part of normal retinal development, and this is necessary for the normal organization of photoreceptor OS morphology (Jablonski, Tombran‐Tink, Mrazek, & Iannaccone, 2000). It is possible that in the absence of PEDF in albinism, photoreceptor OS morphology becomes disorganized, and its ability to elongate is impaired (Jablonski et al., 2000). Potentially, L‐DOPA supplementation is stabilizing the morphology of the photoreceptor OS in albinism, in addition to increasing rod photoreceptor numbers (Jablonski et al., 2000). However, this effect would only be sustained while L‐DOPA is present. Following cessation of L‐DOPA supplementation, an increased rate of cell death might be expected, as the photoreceptor outer segments undergo a continual turnover process.

### 3.10 L‐DOPA supplementation increases photoreceptor end tips (ETPRS) thickness in albino and pigmented mice

There were no significant differences between ETPRS thickness measurements recorded from untreated pigmented (B6) mice and untreated albino (CALBs) mice (see Table 3 for mean values). ETPRS thickness measurements were significantly increased in CALBs supplemented with L‐DOPA from birth (*z* = 3.43, *p* = 0.001) and 15 days PNA (*z* = 3.97, *p* < 0.001), and B6s treated from 15 days PNA (*z* = 10.77, *p* < 0.001) (Table 3, Figure S3). Treatment became less effective with increasing age, until a tendency towards significantly decreased ETPRS thickness measurements emerged in CALBs treated from birth (*z* = −6.74, *p* < 0.001), 15 (*z* = −7.42, *p* < 0.001) and 28 days PNA (*z* = −3.29, *p* = 0.001), and B6 mice treated from 26 days PNA (Figure S3). These observations would be consistent with our hypothesis that L‐DOPA supplementation is temporarily stabilizing the morphology of the photoreceptor outer segments. An initial increase followed by a decrease in ETPRS thickness measurements would be expected, in mice that received a course of L‐DOPA supplementation.

### 3.11 L‐DOPA supplementation increases retinal pigment epithelium (RPE) thickness in albino mice and decreases RPE thickness in pigmented mice

Retinal pigment epithelium thickness measurements were significantly greater in untreated pigmented (B6) mice in comparison with untreated albino (CALBs) mice (*z* = 9.14, *p* < 0.001) (see Table 3 for mean values). Normally, melanin is synthesized from L‐DOPA in the RPE, by melanosomes, which are located in the apically in the RPE cells. Melanin synthesis peaks early in foetal life (coinciding with ocular development) and diminishes with age (Sparrow, Hicks, & Hamel, 2010). This process is impaired/absent in albinism and appears to be manifesting morphologically as a decrease in RPE thickness measurements. RPE thickness measurements were significantly increased in CALBs supplemented with L‐DOPA from 15 days PNA (*z* = 2.4, *p* = 0.016) (Table 3, Figure S3). Significant increases in RPE thickness measurements also became evident with increasing age, in CALBs supplemented with L‐DOPA from birth (*z* = 2.76, *p* = 0.006) (Figure S3). L‐DOPA supplementation would not increase melanin biosynthesis, as there is no functional tyrosinase present in albinism. Perhaps, L‐DOPA is having direct trophic effects on the RPE, through the synthesis of PEDF (Jablonski et al., 2000). Conversely, there was a significant decrease in RPE thickness measurements in B6 mice treated from 15 days PNA (*z* = −3.56, *p* < 0.001) (Table 3, Figure S3). Treatment effect size decreased with increasing age, until significance was lost by 3 months PNA in B6 mice treated from 15 days PNA (*z* = 3.17, *p* = 0.002) (Figure S3).

### 3.12 Retinal layer optical coherence tomography (OCT) measurements are predictive morphological biomarkers for visual function in albino mice

**Figure 4:**
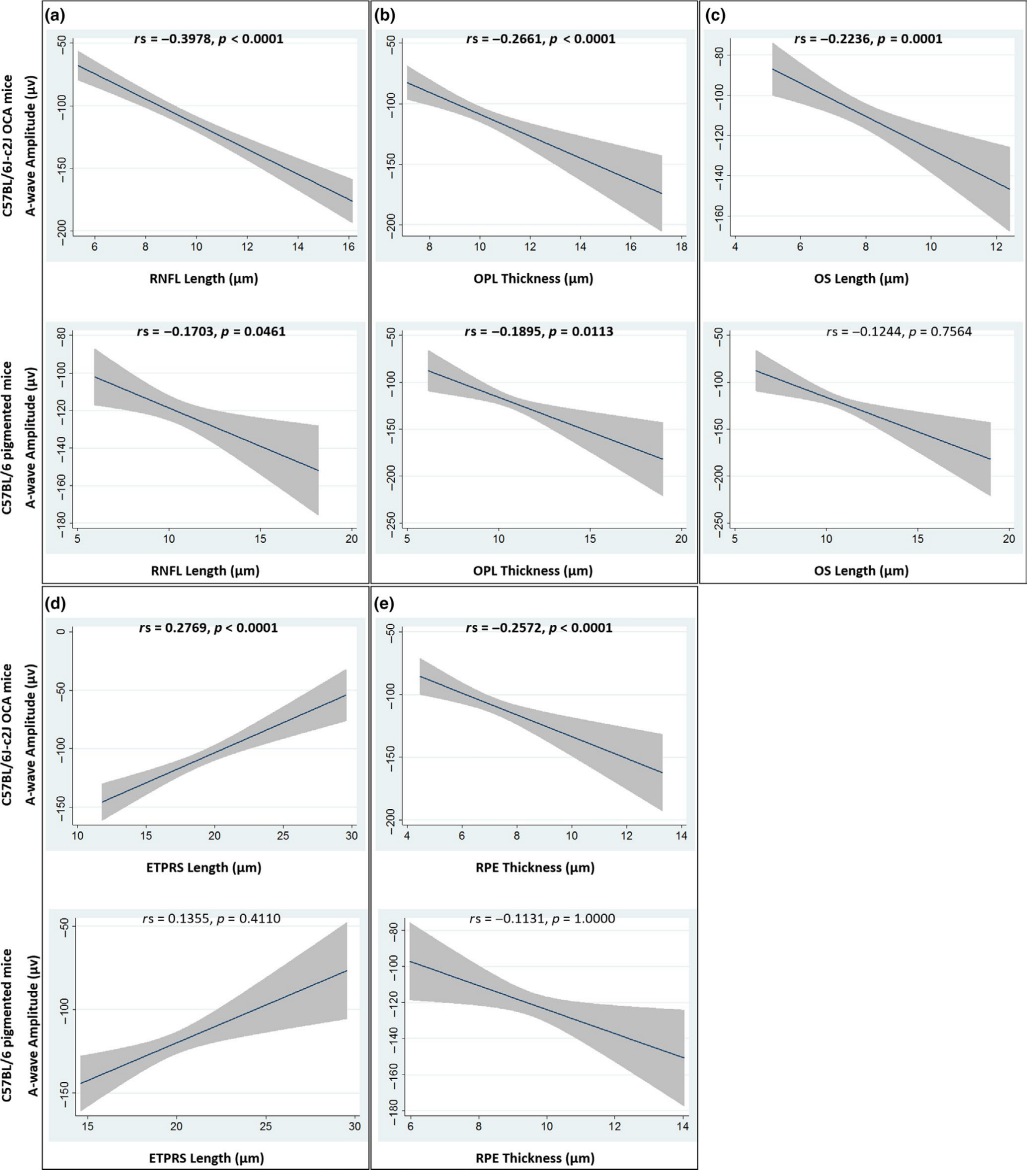
Graphs of the relationship between A‐wave amplitudes and RNFL (a), OPL (b), OS (c), ETPRS (d) and RPE (e) OCT thickness measurements. The best fit linear line together with its 95% confidence interval is shown. Spearman's rho (rs) is shown, together with the Bonferroni adjusted *p* value. Significant values are indicated in bold text. See Figure S4 to view the individual data, colour coded based on treatment group. ETPRS, photoreceptor end tips; OCA, oculocutaneous albinism; OCT, optical coherence tomography; OPL, outer plexiform layer; OS, photoreceptor outer segment; RNFL, retinal nerve fibre layer; RPE, retinal pigment epithelium

**Figure 5:**
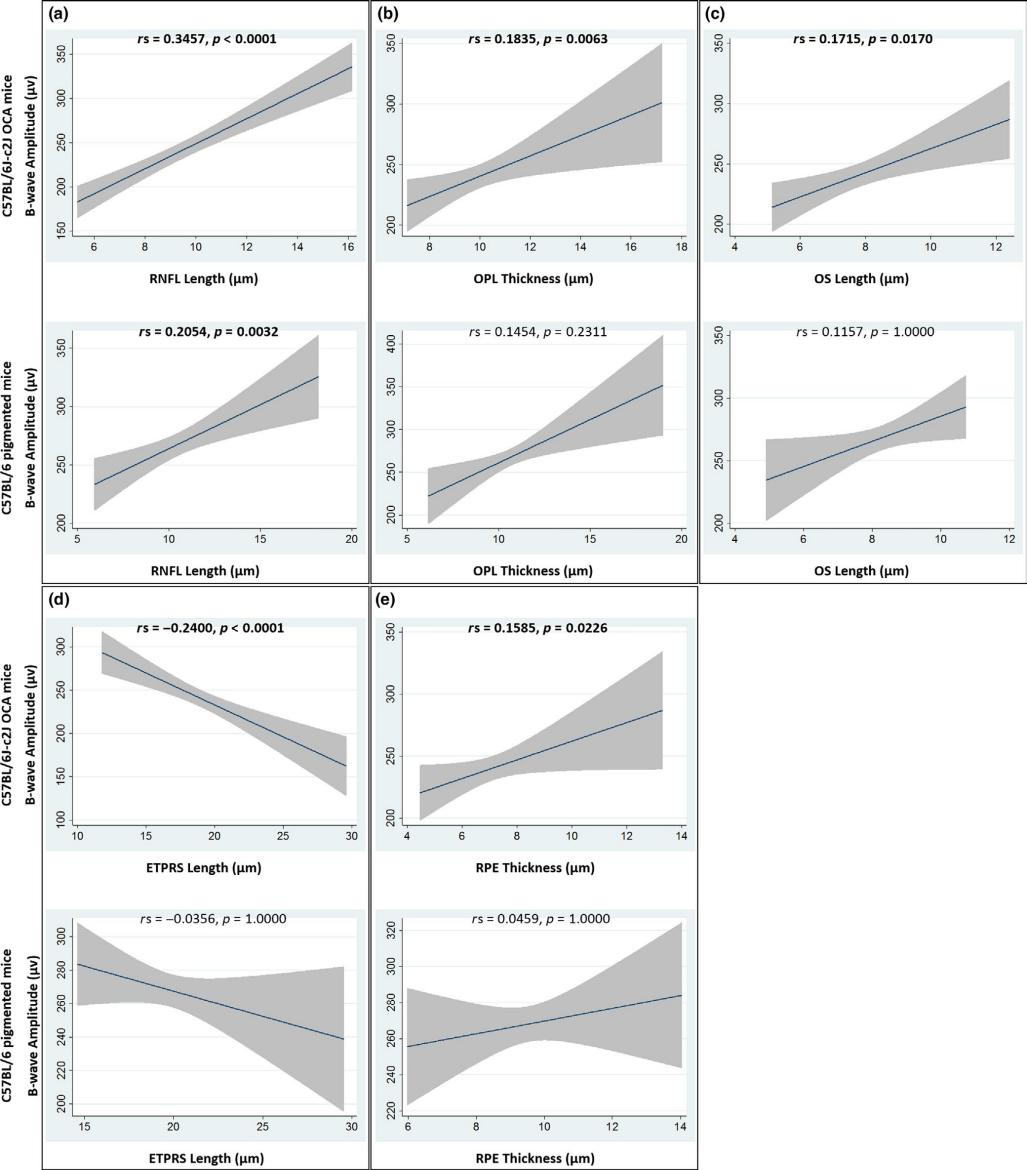
Graphs of the relationship between B‐wave amplitudes and RNFL (a), OPL (b), OS (c), ETPRS (d) and RPE (e) OCT thickness measurements. The best fit linear line together with its 95% confidence interval is shown. Spearman's rho (rs) is shown, together with the Bonferroni adjusted *p* value. Significant values are indicated in bold text. See Figure S4 to view the individual data, colour coded based on treatment group. ETPRS, photoreceptor end tips; OCA, oculocutaneous albinism; OCT, optical coherence tomography; OPL, outer plexiform layer; OS, photoreceptor outer segment; RNFL, retinal nerve fibre layer; RPE, retinal pigment epithelium

Retinal nerve fibre layer (RNFL) OCT thickness measurements have been shown to be reliable surrogate biomarkers for visual function in humans with conditions such as multiple sclerosis (Fisher et al., 2006). The length of the photoreceptor outer segment (OS) has also been correlated with visual acuity in humans with albinism (Thomas et al., 2011). In order to investigate the relationship between retinal layer thickness measurements and A‐ and B‐wave electroretinography (ERG) amplitudes, a Spearman's rank sum correlation was conducted using a Bonferroni correction for multiple comparisons (Table 4). RNFL, outer plexiform layer (OPL), OS, photoreceptor end tips (ETPRS) and retinal pigment epithelium (RPE) measurements correlated significantly with increased A‐ and B‐wave ERG amplitudes (Figures 4 and 5, Figure S4).

**Table 4:** Summary of Spearman's rank sum correlation between A‐ and B‐wave amplitudes and retinal layer thickness measurements, using a Bonferroni correction for multiple comparisons

	CALBs			B6 mice		
	*n*	Spearman's rho	*p*	*n*	Spearman's rho	*p*
a. A‐wave						
RNFL thickness	**426**	**−0.3978**	**<0.0001**	**369**	**−0.1703**	**0.0461**
GCL‐IPL thickness	426	0.1794	0.0089	369	0.1207	0.9152
INL thickness	426	−0.0952	1.0000	369	−0.0562	1.0000
OPL thickness	**426**	**−0.2661**	**<0.0001**	**369**	**−0.1895**	**0.0113**
ONL thickness	426	0.1304	0.3173	369	0.1360	0.3999
IS length	426	0.1448	0.1236	369	0.0656	1.0000
OS length	**428**	**−0.2236**	**0.0001**	369	−0.1244	0.7564
ETPRS thickness	**428**	**0.2769**	**<0.0001**	369	0.1355	0.4110
RPE thickness	**428**	**−0.2572**	**<0.0001**	369	−0.1131	1.0000
b. B‐wave						
RNFL thickness	**426**	**0.3457**	**<0.0001**	**369**	**0.2054**	**0.0032**
GCL‐IPL thickness	426	−0.1495	0.0887	369	−0.1006	1.0000
INL thickness	426	0.0964	1.0000	369	0.0901	1.0000
OPL thickness	**426**	**0.1835**	**0.0063**	369	0.1454	0.2311
ONL thickness	**426**	**−0.1602**	**0.0406**	369	−0.1033	1.0000
IS length	**426**	**−0.1065**	**0.0279**	369	0.0418	1.0000
OS length	**426**	**0.1715**	**0.0170**	369	0.1157	1.0000
ETPRS thickness	**426**	**−0.2400**	**<0.0001**	369	−0.0356	1.0000
RPE thickness	**426**	**0.1585**	**0.0226**	369	0.0459	1.0000

Results highlighted in bold are statistically significant.

4

4.1

In summary, we have shown that post‐natal L‐DOPA supplementation has significant effects on the developing retina in a period of known retinal plasticity. In both albino and control mice, significant increases in the thickness of the RNFL, INL, OPL, ONL and ETPRS layers; significant increases in OS length measurements; and significant decreases in the thickness of the GCL‐IPL retinal layer were seen. RNFL, OPL, OS, ETPRS and RPE layer OCT measurements correlate with retinal function. Significant improvements in retinal function and increases in RPE thickness were only observed in albino mice, while significant increases in IS length were only observed in control mice. We have demonstrated proof of concept for the post‐natal rescue of retinal morphology and function in albinism using oral L‐DOPA supplementation. This appears to be time critical, with the greatest improvements in retinal function observed in mice treated with before 28 days PNA. The effects of L‐DOPA supplementation on retinal morphology are complex and are likely due to a combination of dynamic and interacting effects on cellular proliferation and cell death in the nuclear layers, alterations in photoreceptor numbers and OS structure, and changes in synaptogenesis and dendritic pruning within the plexiform layers in the developing retina. Additional ultrastructural studies are needed in order to confirm that post‐natal L‐DOPA supplementation improves photoreceptor degeneration and/or the aberrant cytoarchitectural changes associated with mouse models of albinism. We are planning further studies to interrogate the detailed molecular, cellular, morphological and functional effects of post‐natal L‐DOPA supplementation on each retinal cell type in albinism and the most efficacious timing and dose of L‐DOPA supplementation in human albinism.

## CONFLICT OF INTEREST

The authors have no conflicts of interest to declare.

## AUTHOR CONTRIBUTIONS

H.L. conceived and designed the experiments, acquired, analysed and interpreted the data, drafted the manuscript and supervised the project; J.S. and H.G. performed the experiments, acquired and analysed the data and reviewed the manuscript; J.E.S. provided technical support and conceptual advice, critically revised the manuscript and helped supervise the project; and A.L. provided technical support and conceptual advice, reviewed the manuscript and helped supervise the project. All authors discussed the results and contributed to the final manuscript.

## Data Availability

The data that support the findings of this study are available from the corresponding author upon reasonable request.
