# Supplementary figures and images for: Oral levodopa rescues retinal morphology and visual function in a murine model of human albinism
Source: Pigment Cell Melanoma Res. 2019 Apr 2;32(5):657–71. doi: 10.1111/pcmr.12782 (PMC6766973; doi:10.1111/pcmr.12782)

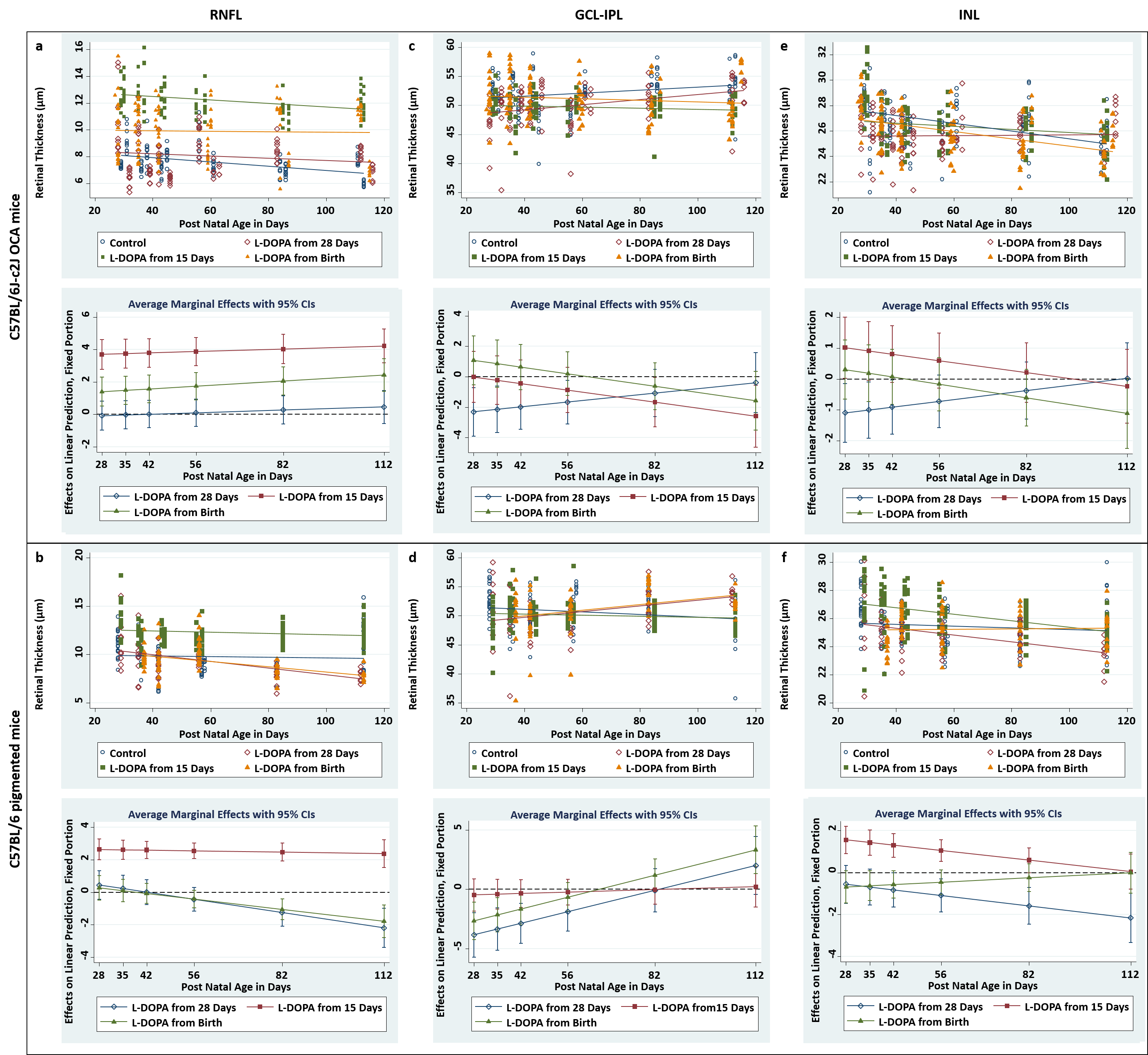

Supplement: Supplementary file 1 [file PCMR-32-657-s001.tif]

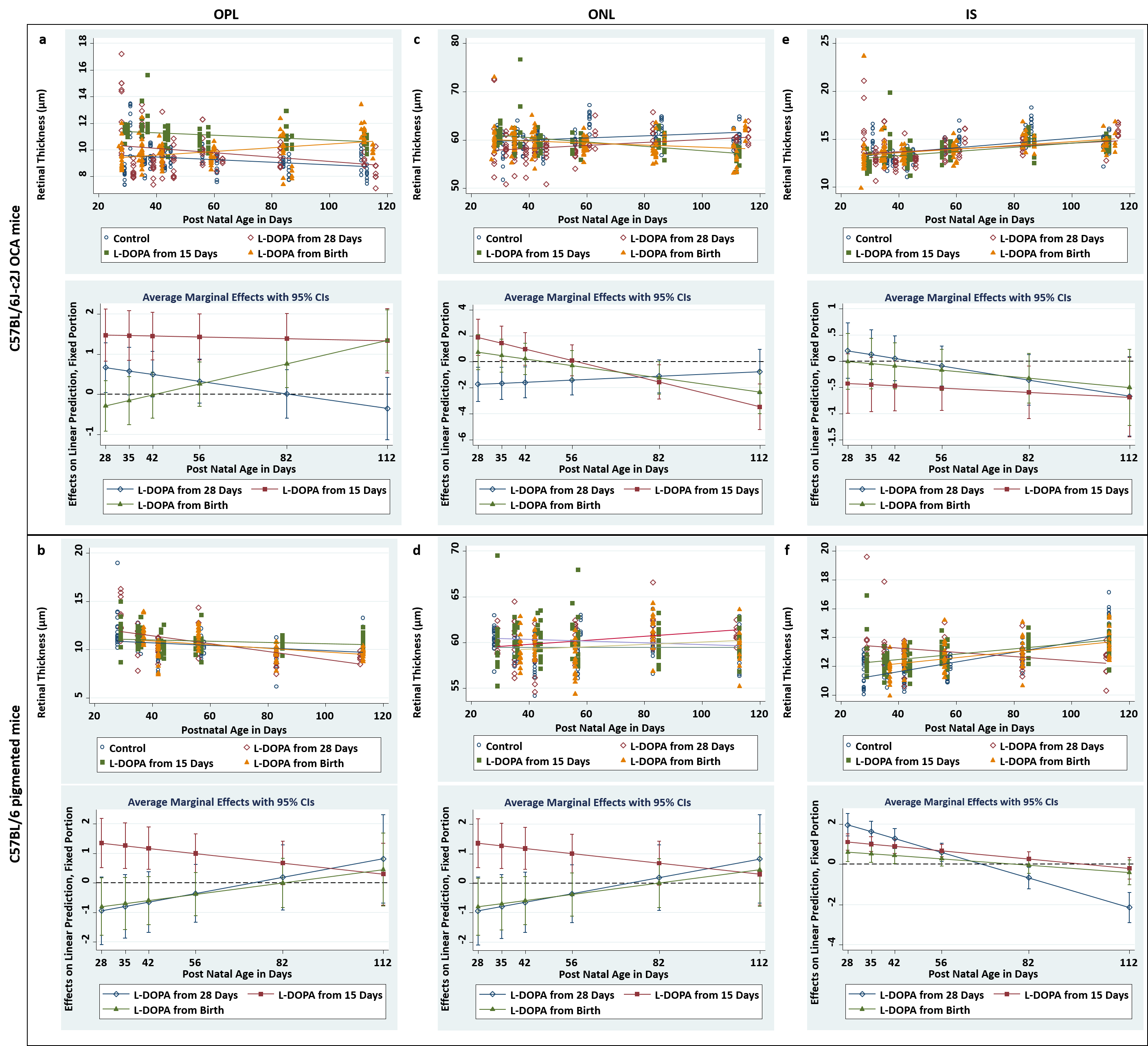

Supplement: Supplementary file 2 [file PCMR-32-657-s002.tif]

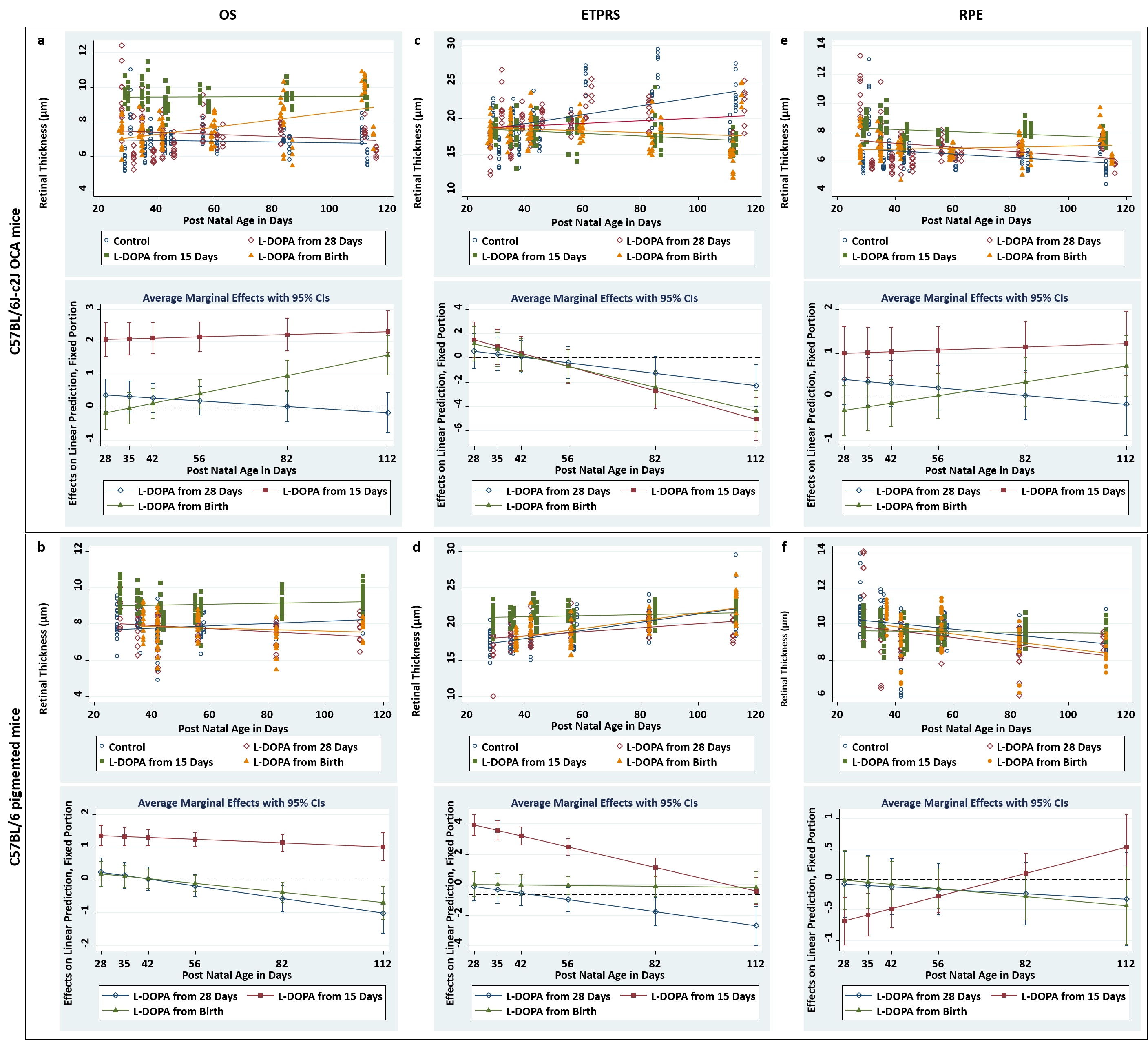

Supplement: Supplementary file 3 [file PCMR-32-657-s003.tif]

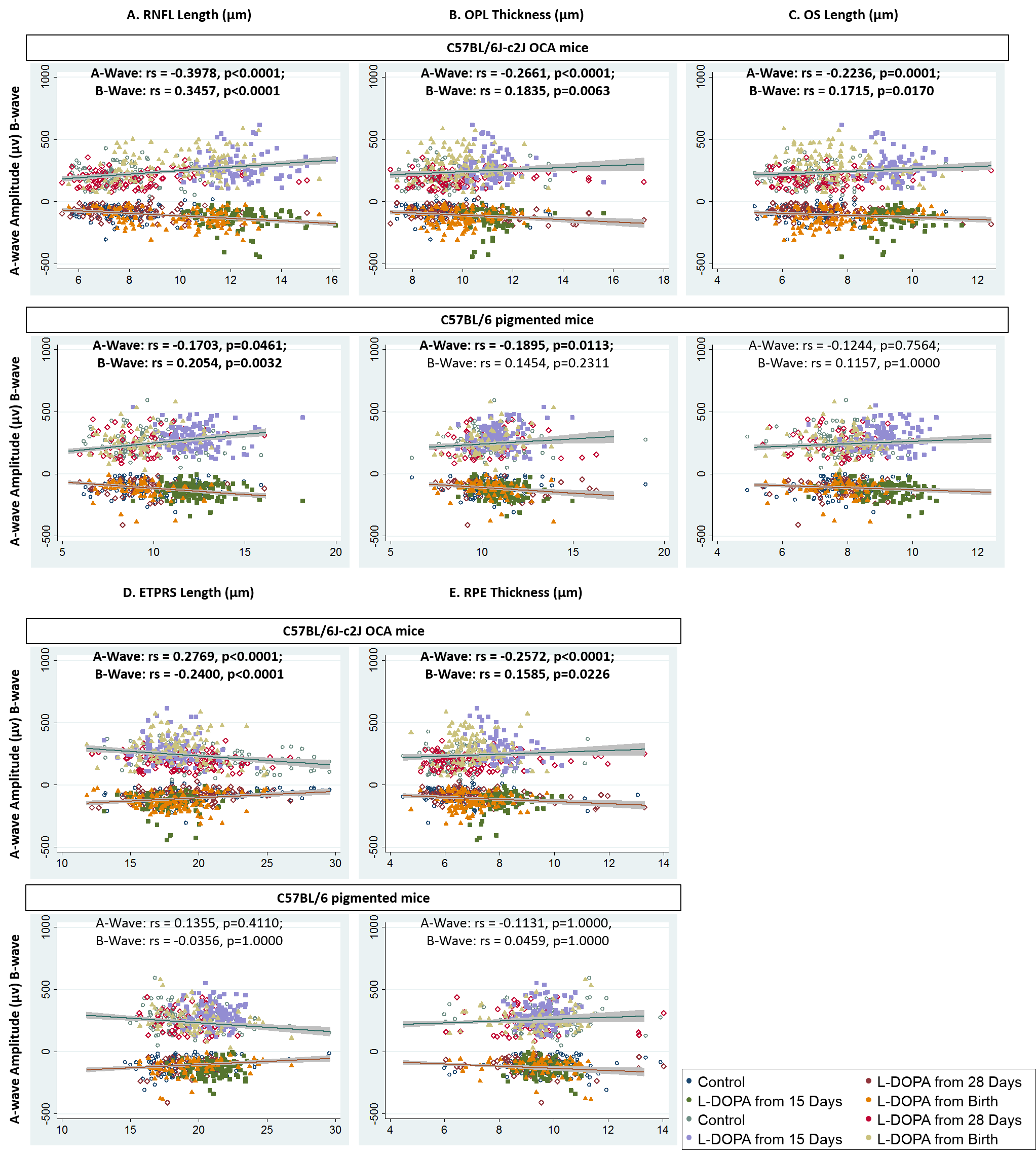

Supplement: Supplementary file 4 [file PCMR-32-657-s004.png]
